# A holistic lifestyle mobile health intervention for the prevention of type 2 diabetes and common mental disorders in Asian women with a history of gestational diabetes: a randomised control trial with 3-year follow-up protocol

**DOI:** 10.1186/s13063-024-08247-x

**Published:** 2024-07-03

**Authors:** Alicia Salamanca-Sanabria, Seaw Jia Liew, Jacqueline Mair, Maria De Iorio, Young Doris Yee Ling, Mya Thway Tint, Yew Tong Wei, Karen Lim, Desmond Ong, Yu Chung Chooi, Vicky Tay, Johan Gunnar Eriksson

**Affiliations:** 1 Singapore Institute for Clinical Sciences, Agency for Science, Technology and Research (A*STAR), Singapore, Singapore; 2 Human Potential Translational Research Program, Yong Loo Lin School of Medicine, National University of Singapore, Singapore, Singapore; 3 Campus for Research Excellence and Technological Enterprise (CREATE), Future Health Technologies, Singapore-ETH Centre, Singapore, Singapore; 4 Saw Swee Hock School of Public Health, National University of Singapore, Singapore, Singapore; 5 Centre for Digital Health Interventions, Department of Management, Technology, and Economics, ETH Zurich, Zurich, Switzerland; 6 Yong Loo Lin School of Medicine, National University of Singapore, Singapore, Singapore; 7 Division of Family Medicine, Department of Medicine, Yoo Loo Lin School of Medicine, National University of Singapore, Singapore, Singapore; 8 Division of Endocrinology, National University Hospital, Singapore, Singapore; 9 Division of Maternal-Foetal Medicine, Department of Obstetrics and Gynaecology, National University Hospital, Singapore, Singapore; 10 Family Medicine Residency Programme, National University Hospital, Singapore, Singapore; 11 Department of Obstetrics and Gynaecology, Yong Loo Lin School of Medicine, National University of Singapore, Singapore, Singapore; 12 Folkhälsan Research Centre, Helsinki, Finland

**Keywords:** Gestational diabetes mellitus, Type 2 diabetes, Depression, Anxiety, Sleep, Women, Preventive healthcare, Digital health, Behaviour change

## Abstract

**Background:**

Women with a history of gestational diabetes mellitus (GDM) are 12-fold more likely to develop type 2 diabetes (T2D) 4–6 years after delivery than women without GDM. Similarly, GDM is associated with the development of common mental disorders (CMDs) (e.g. anxiety and depression). Evidence shows that holistic lifestyle interventions focusing on physical activity (PA), dietary intake, sleep, and mental well-being strategies can prevent T2D and CMDs. This study aims to assess the effectiveness of a holistic lifestyle mobile health intervention (mHealth) with post-GDM women in preventing T2D and CMDs in a community setting in Singapore.

**Methods:**

The study consists of a 1-year randomised controlled trial (RCT) with a 3-year follow-up period. Post-GDM women with no current diabetes diagnosis and not planning to become pregnant will be eligible for the study. In addition, participants will complete mental well-being questionnaires (e.g. depression, anxiety, sleep) and their child’s socio-emotional and cognitive development. The participants will be randomised to either Group 1 (Intervention) or Group 2 (comparison). The intervention group will receive the “LVL UP App”, a smartphone-based, conversational agent-delivered holistic lifestyle intervention focused on three pillars: Move More (PA), Eat Well (Diet), and Stress Less (mental wellbeing). The intervention consists of health literacy and psychoeducational coaching sessions, daily “Life Hacks” (healthy activity suggestions), slow-paced breathing exercises, a step tracker (including brisk steps), a low-burden food diary, and a journaling tool. Women from both groups will be provided with an Oura ring for tracking physical activity, sleep, and heart rate variability (a proxy for stress), and the “HAPPY App”, a mHealth app which provides health promotion information about PA, diet, sleep, and mental wellbeing, as well as display body mass index, blood pressure, and results from the oral glucose tolerance tests. Short-term aggregate effects will be assessed at 26/27 weeks (midpoint) and a 1-year visit, followed by a 2, 3, and 4-year follow-up period.

**Discussion:**

High rates of progression of T2D and CMDs in women with post-GDM suggest an urgent need to promote a healthy lifestyle, including diet, PA, sleep, and mental well-being. Preventive interventions through a holistic, healthy lifestyle may be the solution, considering the inextricable relationship between physical and psychological health. We expect that holistic lifestyle mHealth may effectively support behavioural changes among women with a history of GDM to prevent T2D and CMDs.

**Trial status:**

The protocol study was approved by the National Healthcare Group in Singapore, Domain Specific Review Board (DSRB) [2023/00178]; June 2023. Recruitment began on October 18, 2023.

**Trial registration:**

ClinicalTrials.gov NCT05949957. The first submission date is June 08, 2023.

**Supplementary Information:**

The online version contains supplementary material available at 10.1186/s13063-024-08247-x.

## Introduction

### Background and rationale {6a}

Individuals, families, and societies worldwide are affected by diabetes. According to recent reports, one in ten adults has diabetes worldwide, accounting for 10.5% of the global population [1, 2]. Globally, it is projected to be 700 million people with diabetes, with women being 343 million by 2045 [2]. Diabetes ranks ninth for women and eighth for men in the global ranking of disability-adjusted life years (DALYs) [3, 4]. There is a parallel increase in obesity and type 2 diabetes (T2D) in the general population, as well as a rise in gestational diabetes mellitus (GDM) incidence [5]. GDM affects between 1 and 30% of pregnancies worldwide [6] and is particularly prevalent among Asian women [7], showing distribution in the Middle East (8.8–20.0%), Southeast (9.6–18.3) and Western Pacific (4.5–20.3%), respectively [6]. A recent report in Singapore shows that GDM prevalence is 23.5% [8]. GDM can cause pregnancy and birth complications and long-term chronic conditions such as cardiometabolic disorders and T2D in both mother and offspring [9, 10]. Compared to women with a normoglycemic pregnancy, women with a history of GDM are more likely to develop T2D [11]. The prospective birth cohort study in Singapore, Growing Up in Singapore Towards Healthy Outcomes (GUSTO), showed that the risk of developing T2D is 12-fold higher in Singaporean women 4–6 years after their GDM diagnosis [12].

Risk factors for T2D among those with a history of GDM included greater pre-pregnancy Body Mass Index (BMI), excessive weight gain, unhealthy dietary patterns, physical inactivity, and a short period of lactation [13]. Post-natal risk, such as missing medical assistance in the continuum of GDM care after delivery, could be another risk for progression to T2D among Asian mothers with a history of GDM [7]. In terms of the pathophysiology, GDM has several similarities to T2D, namely impaired insulin sensitivity and dysfunction of the β-cell caused by the metabolic stress of pregnancy [5, 14].

Besides T2D and cardiometabolic diseases, current literature suggests that women with a history of GDM also have a higher risk of developing common mental disorders (CMDs) (depression, anxiety) [15, 16]. CMDs are the most common morbidity in the peripartum (during pregnancy and up to 1 year following delivery). Approximately one in five women develop CMDs during pregnancy or in the year following delivery [17], which is associated with adverse maternal and foetal outcomes as well as emotional and behavioural difficulties in the offspring [18]. Likewise, poor quality of sleep associated with gestational diabetes can pose significant health risks to both mothers and their newborns [19–21]. Previous studies have shown that women with GDM have shorter sleep time, less efficient sleep, and more sleep disorders and daytime dysfunction [19]. The evidence has been demonstrated that sleep disturbances can impair glucose metabolism by affecting insulin sensitivity and β-cell function, which is also associated with obesity and depression [20]. Thus, women with a history of GDM also may present poor sleep quality and CMDs. However, effective interventions that aim to improve these women’s mental well-being and sleep quality remain scarce.

A systematic review and meta-analysis about the prevention of T2D in women with previous GDM has shown that lifestyle interventions produced only a borderline reduction in T2D risk (RR 0.75, 95% CI: 0.55–1.03) [22]. The interventions did not focus on modifiable risk factors like sleep and mental well-being, which could have been addressed to improve the outcome. Observational studies suggest that approximately 45% of GDM cases might be preventable by the adoption of a healthy diet and increased physical activity (PA), including psychosocial factors (stress, anxiety, depression interventions) [6, 23–25]. However, most preventative studies are undertaken in Western countries [21], and it is challenging to directly apply these strategies in Asian countries due to differences in demographics, socioeconomics, ethnicity, and cultural factors [7]. A recent systematic review study concluded that in most of the studies, the evidence-based intervention might not be culturally appropriate for the prevention of T2D in women with a history of GDM [13].

Postpartum women often report difficulty engaging with lifestyle management interventions due to several barriers [26, 27], including multiple face-to-face sessions, travel distances, time constraints, and cost are some factors [26, 28] that may be alleviated by technological advancements [29]. Thus, lifestyle interventions delivered through mobile health (mHealth) interventions represent a feasible, affordable and scalable solution for the prevention of T2D and CMDs among women with a history of GDM [30]. While several mHealth lifestyle intervention studies have been conducted to prevent NCDs [31, 32] and CMDs [33] few have taken a holistic approach to improving health and mental wellbeing. The inextricable link between physical and psychological health [2, 4, 22–24] highlights the combined effects of holistic interventions integrating body and mind may have a greater total effect than any single intervention in preventing T2D and CMD [34]. The LvL UP is a scalable holistic mHealth intervention to prevent NCDs and CMDs in Asian populations [35]. The intervention has been informed by leading evidence- and theory-based frameworks in mental health [36] and behaviour change [36] to deliver motivational interviewing-inspired digital coaching via conversational agent and behavioural tools centred around three core pillars: Move More, Eat Well, Stress Less. The intervention has undergone rigorous development and refinement, including feasibility and user-centred design studies (unpublished data) to assess its technical feasibility and acceptability, but has not yet been tested for effectiveness.

### Objectives {7}

This study aims to assess the effectiveness of a scalable smartphone-delivered holistic lifestyle coaching intervention for the prevention of T2D and CMDs with post-GDM Asian women from the community setting in Singapore. We have five specific objectives:

- Objective 1: To carry out a 1-year randomised control trial (RCT) followed by a 3-year follow-up period with post-GDM women from the community setting in Singapore.
- Objective 2: To examine the potential impacts of the proposed intervention on the health and mental well-being of women and their children.
- Objective 3: To determine the diabetes risk of the participants over a 3-year follow-up period.
- Objective 4: To explore the potential economic impacts of the proposed intervention (e.g. healthcare expenditures)
- Objective 5: To study the importance of gut microbiota and epigenetic factors in changes in glucose metabolism.

### Trial design {8}

This study is a 1-year randomised controlled trial (RCT) with 3 years of follow-up. We will conduct a two-arm single parallel assignment individually randomised equal allocation control trial on *n* = 400 women with a history of GDM. It will compare the aggregate effects of the Intervention group (receiving a holistic lifestyle mHealth intervention (LvLUP App), Sleep tracker, Oura ring/Oura App and psychoeducation to promote healthy lifestyle (Happy App)) and (comparison group (Happy App (psychoeducation only), Oura ring and Oura App) at 26/27 weeks, and 1-year visit. After completing the 1-year RCT period, both groups will be followed up in years 2, 3, and 4. We hypothesise that the intervention will reduce the risk of T2D/pre-diabetes and CMDs in post-GDM women.

## Methods: participants, Interventions, and outcomes

### Study settings {9}

Participants are women with a history of GDM from a large community setting in Singapore.

### Eligibility criteria {10}

All eligible females in this study will be (a) women between 21 and 45 years old; (b) with a history of GDM (at least 1 year and no more than 10 years); (c) Chinese, Malay, or Indian ethnicity; (d) BMI between 18.5 and 35 kg/m^2^; (e) not planning to conceive in the next 1 year; (f) not performing exclusive breastfeeding during the study period; (g) owners of a smartphone compatible with the study mobile Apps; (h) proficient in the English language; (j)willing to comply with study protocol; and (k) able to provide written informed consent.

Exclusion criteria include (a) current or previous diagnosis of diabetes (type 1 or 2), except GDM; (b) currently pregnant; (c)given birth within the last 12 weeks; (d) severely limited mobility (e.g. wheelchair-bound, require long-term walking aid, etc.); (e) diagnosed with malnutrition or eating disorder; (f) diagnosed with cancers, unstable heart diseases, severe kidney disease, severe liver disease; (g) diagnosed with severe insomnia, unstable mental conditions, dementia, or cognitive impairment; (h) experienced alcohol or drug abuse; (i) currently having medications known to influence glucose metabolism (e.g. peroral corticosteroids); and (j) currently participating in a concurrent clinical trial or lifestyle intervention study.

### Who will solicit informed consent? {26a}

Consent procedures were approved by the Domain Specific Review Board (DSRB) of the National Healthcare Group in Singapore. All potential subjects are required to sign the informed consent in order to participate in the study.

### Additional consent provisions for collection and use of participant data and biological specimens in ancillary studies {26b}

There are no additional consent provisions for collecting or using biological samples. If we decide to do an ancillary study in the future, we will seek prior consent of participants to be re-contacted and re-consent for the ancillary study following DSRB approval.

## Interventions

### Explanation for choice of comparators {6b}

mHealth may provide practical and scalable support to women adopting and maintaining a healthy lifestyle. The effectiveness of mHealth interventions targeting lifestyle behaviours has been demonstrated [31, 34, 37]. More recently, studies have supported that prevention and promotion request more than one pillar, meaning that holistic lifestyle behaviours, such as PA, diet, sleep, and mental well-being, are more effective in parallel [34]. Studies have shown that education alone is insufficient to support individuals in changing their lifestyles, and it is important to incorporate techniques and behavioural strategies that facilitate and measure the effectiveness of the interventions [31, 34, 37]. Therefore, we propose to compare two mHealth interventions, the intervention group (LvL UP App, Happy App, and Oura Ring) and the comparison group (HAPPY App and Oura Ring). The LvL UP App is based on evidence-based strategies and interactive tools developed in Singapore [35]. The HAPPY App is available to every eligible participant (both groups) in the trial; it provides public holistic health recommendations and displays participants’ health data measured during study visits over time. Also, the Oura ring is a sleep tracker that includes heart rate variability, and a step tracker will be provided to both groups.

### Intervention description {11a}

The comparison and intervention group will be assigned to holistic lifestyle mHealth interventions. The comparison group (Happy App and Oura ring) will receive only psychoeducation to promote a healthy lifestyle through the HAPPY App and sleep tracking using an Oura ring and associated App. The Happy App was developed by the Singapore Institute for Clinical Sciences (SICS); it encompasses only educational content on lifestyles based on resources from the Singapore Health Promotion Board, including information about diet intake, physical activity, sleep, and mental well-being. The HAPPY app also displays health outcome data collected during the study visits (e.g. body weight, blood pressure, fasting glucose, and 2-h 75 g OGTT). The Oura Ring is an activity-tracking wearable that monitors physical activity, sleep, and heart rate variability, which can be synced with the Oura App.

The intervention group (LvL UP App, Happy App, and Oura Ring) will receive the same Happy App, Oura Ring and Oura App, with the additional LvL UP App version 2.0. The LvL UP App has been developed by the Future Health Technologies Programme at the Singapore ETH-Centre (SEC) for iOS and Android. It is designed to be culturally relevant in Singapore and is based on evidence-based behavioural strategies [35]. A separate paper[35] describes the conceptual model, development process, and characteristics of LvLUP version 1.0. In this study, we will use an updated 2.0. version. The LvL UP App 2.0 is a smartphone-delivered holistic lifestyle intervention focusing on three core pillars: *eat well* (diet), *move more* (PA), and *stress less* (mental well-being), including sleep content in each pillar delivered through a coaching health and tools paradigm.

#### LvL UP App: coaching health

The participants assigned to LvL UP will receive weekly motivational interview-inspired coaching sessions from a digital coach (conversational agent; CA) on topics related to the core intervention pillars. Coaching sessions are text-based dialogues between the participant and the CA based on predefined rules, and each session lasts between pillars, which are recommended in a predefined order, although participants can choose the order and topic if they wish. There are eight coaching sessions per pillar, which are recommended in a predefined order, although participants can choose the order and topic if they wish. The participant starts the intervention with a welcome dialogue, which introduces the LvL UP App and assesses the participant’s needs in each of the core pillars using the Patient Health Questionnaire-4 (PHQ-4) [38], a Modified Food Frequency Questionnaire (FFQ) based on dietary recommendations, and the International Physical Activity Questionnaire-Short Form (IPAQ-SF) [39]. Based on the participant’s needs, a starting pillar is recommended, but the participant can still choose the pillar they wish to focus on. Following the welcome dialogue, the participants complete two short sessions to identify their perceived values and strengths, which are later reflected to the participant by the CA during coaching sessions.

#### LvL UP App: tools

Additionally, the LvL UP App includes different tools that complement each pillar, including a set of self-regulatory behaviour change techniques, actionable habit-forming suggestions, and a slow-paced breathing training mini-game. The self-regulatory tools include a low-burden image tracking food diary (*MakanMemo*), a step tracker (*StepLah!*) that displays steps collected by Apple Health (iOS) or GoogleFit (Android) and allows users to set and track step goals, including brisk steps (120 steps/min or higher), and a *Journal* with different templates inspired by cognitive behavioural therapy to support mental well-being. Life Hacks are actionable tips for improving healthy habits and are designed to be easily executed during the daily routine. They operate on the premise that taking small, concrete actions can accumulate and lead to noticeable changes over time. Finally, Breeze is a slow-paced breathing training mini-game that uses breathing data collected by the smartphone microphone to move a sailboat along a river in real time [40].

The intended use of the LvL UP intervention is to complete weekly coaching sessions and use the tools daily. Gamification is built into the App whereby a puzzle piece is awarded for completed activities to fill up the LvL UP Shield. Once the shield is filled, the participant progresses to the next level and the shield resets. There are three levels in total. The LvL UP App delivers reminders and prompts (push notifications) related to intervention actions to encourage the participant to complete the intervention as intended.

### Criteria for discontinuing or modifying allocated interventions {11b}

The participant will be withdrawn from the study if she becomes pregnant during the 1-year intervention period. If a participant becomes pregnant during the 3-year follow-up period, she will skip the follow-up visit for the year and return to the subsequent follow-up visit(s) post-pregnancy. In the same way, if a participant is diagnosed with T2D by her doctor during the 1-year intervention period, she will be invited to complete the endpoint study visit measures before being withdrawn from the study. If the participant is diagnosed with T2D by the study team during the follow-up visits, she will be removed after completing that visit.

### Strategies to assess and improve adherence to interventions {11c}

User adherence in mHealth interventions varied based on user-related, content-related, and technology (personalise remainders, user-friendly, technically stable App, personal support, and gamification features) related barriers and facilitators of engagement [41, 42]. The intervention group [LvL UP] will receive notifications and reminders about the coaching session’s date and time, daily random lifehack, and motivational messages to engage with the LvL UP App. Additionally, a dashboard link to monitor the participant’s live activity regarding last-time interaction, tools used, and the current level is considered to reach up to support adherence with the participants. Thus, the following three strategies will be implemented: calling participants who have not interacted for 2 weeks with the LvL UP App after baseline; texting the participants who have low interaction (e.g. using the App once/twice for 2 weeks), and sending automatic SMS 3, 5 and 7 weeks after the baseline visit to encourage them to use the App.

The HAPPY App will detect the number of logged entries and the last interaction with the app. A manual schedule reminder will be sent by the App if the participant has not read the suggested articles or documents.

### Relevant concomitant care permitted or prohibited during the trial {11d}

The participants will be excluded if they have enrolled in a concurrent lifestyle intervention research study during the study. However, trial participants can receive concomitant treatment (e.g. psychotherapy, pharmacology), which will be assessed during the follow-up period.

### Provisions for post-trial care {30}

Access to the Happy App will continue for the 3-year follow-up period after the trial for all the participants.

### Outcomes {12}

Table 1 shows the outcomes and timeline of the study. The primary outcome will be the onset of T2D/confirmed by a 2-h 75 g oral glucose tolerance test (OGTT) over a 4-year study period. OGTT will be done at the screening visit, year 1 visit, and follow-up visits in years 2, 3, and 4. Secondary outcomes include (a) onset of impaired fasting glucose and impaired glucose tolerance; (b) changes in cardiometabolic variables (body weight, HbA_1c_, insulin, blood lipids, blood pressure), and (c) changes in women’s body composition as assessed by bioelectrical impedance analysis (BIA). Secondary measures will be taken at baseline visits, middle point, year 1, and 2, 3, and 4-year follow-up period.

**Table 1:** Outcomes and timeline

Assessments	Screening visit	Baseline visit	Midpoint visit (26/27 weeks)	Year 1 visit	Year 2 visit (follow-up)	Year 3 visit (follow-up)	Year 4 visit (follow-up) endpoint
Lab tests							
OGTT test	X				X	X	X
Height, weight and blood pressure measurements	X	X	X	X	X	X	X
Bio-sample collection: buccal swab, saliva, and stool samples		X		X			X
BIA		X		X	X	X	X
Fasting blood draw		X	X	X	X	X	X
Questionnaires							
Demographics	X			X			
GDM and family history of diabetes	X						
Spouse and child health	X			X			
Lifestyle and dietary	X			X			
Diabetes awareness	X			X			
14-day e-Diary recording		X	X	X	X	X	X
SF-36		X	X	X	X	X	X
STAI		X	X	X	X	X	X
BDI-II		X	X	X	X	X	X
CERQ		X	X	X	X	X	X
MEQ		X	X	X	X	X	X
PSQI		X	X	X	X	X	X
SHS		X	X	X	X	X	X
HLQ		X	X	X	X	X	X
PSS-4		X	X	X	X	X	X
WHO-5		X	X	X	X	X	X
Child-related questionnaires							
BISQ-R-SF		X		X			
CSHQ		X		X			
StimQ toddler/preschool		X		X	X	X	X
ASQ-3		X		X	X	X	X
BRIEF-2		X		X	X	X	X
BASC-3-BESS Parent Form		X		X	X	X	X
Questionnaires administrated within the LvLUP App (intervention group only)							
PHQ-4		x	x				
IPAQ-SF		x	x				
MFFQ		x	x				

*OGTT* 2-h 75 g oral glucose tolerance test, *BIA* Bioelectrical impedance analysis, *SF-36* short-form health survey, *STAI* The Stare-Trait Anxiety Inventory, *BDI-II* Beck Depression Inventory, *CERQ* Cognitive Emotion Regulation Questionnaire, *MEQ* Morningness-Eveningness Questionnaire, *PSQI* The Pittsburgh Sleep Quality Index, *SHS* Subjective Happiness Scale, *HLQ* Health Literacy Questionnaire, PSS-4 Perceived Stress Scale 4, *WHO-5* World Health Organization Wellbeing Index, *BISQ-R-SF* Brief Infant Sleep Questionnaire-Revised Short Form, *CSHQ* Children’s Sleep Habits Questionnaire, StimQ toddler/preschool Cognitive Home Environment Questionnaire, *ASQ-3* Ages & Stages Questionnaires-3, *BRIEF-2* Behaviour Rating Inventory of Execute Functions-2, *BASC-3-BESS Parent Form* Behavioral and Emotional Screening System, *PHQ-4* Patient Health Questionnaire, *IPAQ-SF* The International Physical Activity Questionnaire-Short Form, *MFFQ* Modified Food Frequency Questionnaire

Additionally, we will assess changes in women’s mental well-being by completing the following questionnaires at all study visits:

*Beck Depression Inventory* (BDI-II): A 21-item self-reported rating inventory using a 3-point Likert scale that measures symptoms and severity of depression, classified as follows: minimal (0–13); mild (14–19); moderate (20–28); and severe (29–63). The BDI-II internal consistency as measured by Cronbach’s alpha ranges from 0.87 to 0.93 [43].

*State-Trait Anxiety Inventory* (STAI): A 40-item self-report questionnaire using a 4-point Likert scale. The STAI measures two types of anxiety — state anxiety and trait anxiety, which are classified as no or low anxiety (20–37), moderate anxiety (38–44), and high anxiety (45–80). Internal consistency coefficients for the scale have ranged from 0.86 to 0.95 [44].

*Cognitive Emotion Regulation Questionnaire* (*CERQ*): A 36-item self-report measure designed to identify the cognitive emotion regulation strategies (or cognitive coping strategies) someone uses after having experienced adverse events or situations. It is commonly used to assess individual differences in the cognitive regulation of emotions in response to stressful, threatening, or traumatic life events. Internal consistency ranges from 0.72 and 0.83 [45].

*Subjective Happiness Scale (SHS):* A 4-item self-report measure to assess an individual’s overall happiness. Internal consistency ranges from 0.79 to 0.94 [46].

*WHO-5 Wellbeing* Index (*WHO-5*): A 5-item self-reported questionnaire that aims to assess mental wellbeing over the last 2 weeks. The WHO-5 shows reliability across countries between 0.86 and 0.96 [47].

*Perceived Stress Scale* (*PSS-4*): A brief self-report measure of the extent to which recent life events are considered stressful. The PSS-4 has demonstrated acceptable criterion validity and internal consistency (*α* = 0.72) [48].

*The short Form Health Survey* (*SF-36*): Indicates overall health status; it aims to assess the impact of clinical and social interventions on quality of life in several domains. The SF-36 has shown a reliability of 0.90 [49].

*Health Literacy Questionnaire* (*HLQ*): The HLQ examines an individual’s understanding, access and engagement with health information and health services. The HLQ Cronbach’s alpha has shown an internal consistency of 0.87 [50].

Moreover, changes in women’s sleep will be assessed using the following measurements:

*Morningness-Eveningness Questionnaire* (*MEQ*): A 19-item MEQ aims to assess morningness and eveningness where questions are asked preferentially, i.e. asking respondents to indicate when, for example, they would prefer to wake up or start sleep, rather than when they do. MEQ internal consistency is 0.82 [51].

*Pittsburgh Sleep Quality Index* (*PSQI*): Measures the quality and patterns of sleep-in adults. It differentiates “poor” from “good” sleep quality by measuring seven areas (components): subjective sleep quality, sleep latency, sleep duration, habitual sleep efficiency, sleep disturbances, use of sleeping medications, and daytime dysfunction over the last month. The PSQI Cronbach’s alpha has shown good internal consistency ranging from 0.64 to 0.83 [52].

Furthermore, changes in the health and well-being of the women’s children will be collected. Only children born during a GDM pregnancy will be considered in this study. The following measurements will be used:

*Cognitive Home Environment Questionnaire* (*StimQ*): The StimQ-toddler (age 12–35 months)/StimQ-pre-school (for age 36 – 72 months) is designed to identify the different types of toys and games that children have in the home and the kinds of activities that mother and child do together [53]. The StimQ has demonstrated good internal and external reliability (*α* = 0.85) [54].

*Ages & Stages Questionnaires-3* (*ASQ-3*) (1–66 months): The ASQ-3 development screening tool assesses children’s communication, autonomy, compliance, adaptive functioning, affect, and interaction with people. ASQ-3 is a reliable and valid instrument with a test–retest reliability of 92%, a sensitivity of 87.4% and a specificity of 95.7% [55].

*Behaviour Rating Inventory of Executive Functions-2 (BRIEF-2) parent form*: BRIEF-2 is designed for use in medical and educational settings to estimate the global executive function of children/youth and determine whether a comprehensive assessment is appropriate (age 5–18 years). BRIEF-2 has shown reliability coefficients ranging between 0.8 and 0.9 [56].

*Behavioural and Emotional Screening System (BASC-3-BESS) Parent Form*: This screens children and adolescents’ behavioural and emotional strengths and weaknesses (age 3–18 years 11 months). BASC-3 BESS has high internal consistency at 0.80 [57].

Changes in the child’s sleep will be assessed at the baseline visit and the first-year visit. The following is a description of child sleep questionnaires:

*The Brief Infant Sleep Questionnaire-Revised Short Form* (*BISQ-R-SF*): A parent-reported toddler (0–3 years) sleep over the prior 1 week, assessing sleep patterns, ecology, and parental perceptions of sleep. The BISQ-R indicates excellent reliability at 0.80 [58].

*Children’s Sleep Habits Questionnaire (CSHQ) Short Form (SF)version*: The SF-CSHQ assesses the frequency of children’s behaviours associated with common paediatric sleep difficulties for children between 48 and 72 months. The SF-CSHQ has been validated, showing Internal consistency coefficients ranging from 0.90 to 0.94 [59].

After completing the 1-year RCT period, both groups will be followed up for 3 years. During the follow-up visits, body measurements, OGTT, bio-sampling, and data collection will be conducted, and the questionnaires listed above.

Additionally, the intervention group will also complete the following questionnaires through the LvL UP App:

*Patient Health Questionnaire-4* (*PHQ-4*): A 4-item self-report measure that assesses anxiety and depression symptoms. Each item is scored on a 4-point scale (0–3), and scores range from 0 to 27. The score can be used to describe a patient’s symptoms in one of the five categories: none (0–2), mild (3–5), moderate (6–8), and severe (9–12), The PHQ-4 has been shown to have good internal reliability (Cronbach alpha = 0.82) [38].

*Modified Food Frequency Questionnaire* (*MFFQ*): A 7-item self-report checklist of food and beverages with a frequency response section based on My Healthy Plate Singapore[60].

*The International Physical Activity Questionnaire-Short Form* (*IPAQ-SF*): A self-report measure assesses the type of intensity of physical activity and sitting time[39]. The IPAQ-SF has shown a moderate internal consistency of 0.647 [61, 62].

#### Screening survey overview and follow-up

The screening surveys will include questions of 8 types: (i) socio-demographics, (ii) GDM and family history of diabetes, (iii) medical history, (iv) lifestyle, (v) dietary, (vi) diabetes awareness, (vii) paternal demographics and health and (viii) child demographics and health (see Table 2). Those surveys will also be completed in baseline and 1-year visits (see Table 1).

**Table 2:** Screens survey variables overview

(i) *Socio-demographics*: Age, ethnicity, marital status, highest level education, nationality, residency status, occupation, number of children, accommodation type, total monthly household income
(ii) *GDM and family history of diabetes*: First diagnosis of GDM, last diagnosis of GDM, total of GDM diagnosis, number of births, number of babies, mode of delivery, preterm delivery, insulin during pregnancy, medication during pregnancy, medication to treat GDM, maternal complications, neonatal complications, breastfeeding
(iii) *Medical history*: Past and current medical conditions or surgeries, paternal medical history, maternal medical history, siblings’ medical history
(iv) *Lifestyle*: What is considered a healthy lifestyle, barriers to achieving a healthier lifestyle, coping strategies during stress, following specific diet(s), reasons for choosing the diet(s), alcohol consumption, tobacco consumption, eating habits, physical activity, sleep patterns, stress level, changes in lifestyle in the past 3 months, planning to make changes in any lifestyle behaviours in the future, use of activity tracking wearable, use of lifestyle mobile interventions, currently joining a fitness or wellness program
(v) *Dietary*: Dietary habits, oil/fat used for cooking, consumption of coffee/tea or other beverages, rice, fish, red meat, pork, chicken, salt, soya, and vegetables
(vi) *Diabetes awareness*: knowledge about diabetes, GDM, its causes and risks, last time checked blood sugar level, HbA1c test, fasting, 2-h OGTT
(vii) *Paternal demographics and health*: Age, ethnicity, highest level of education, nationality, residency status, occupation, overall height, overall weight, paternal medical history, maternal medical history, siblings’ medical history
(viii) *Child demographics and health*: Age, ethnicity, sex, weight at birth, height at birth, head circumference, education level, medical condition(s), preterm birth, hospitalizations

### Participants timeline {13}

**Fig. 1:**
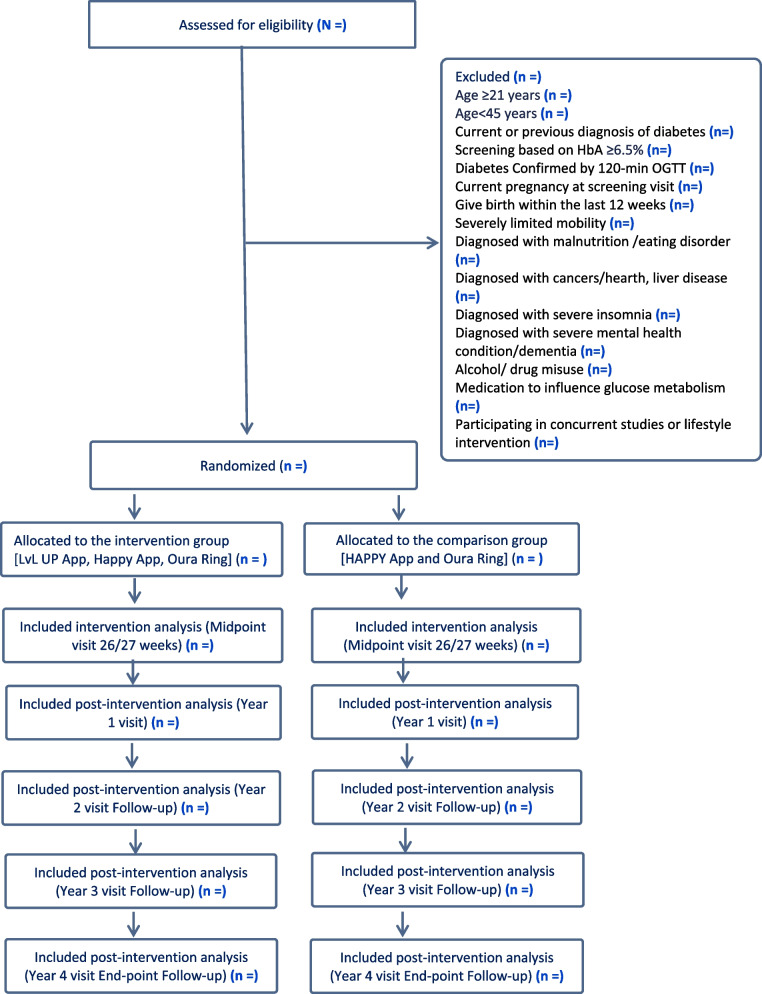
Participant flow consort

**Fig. 2:**
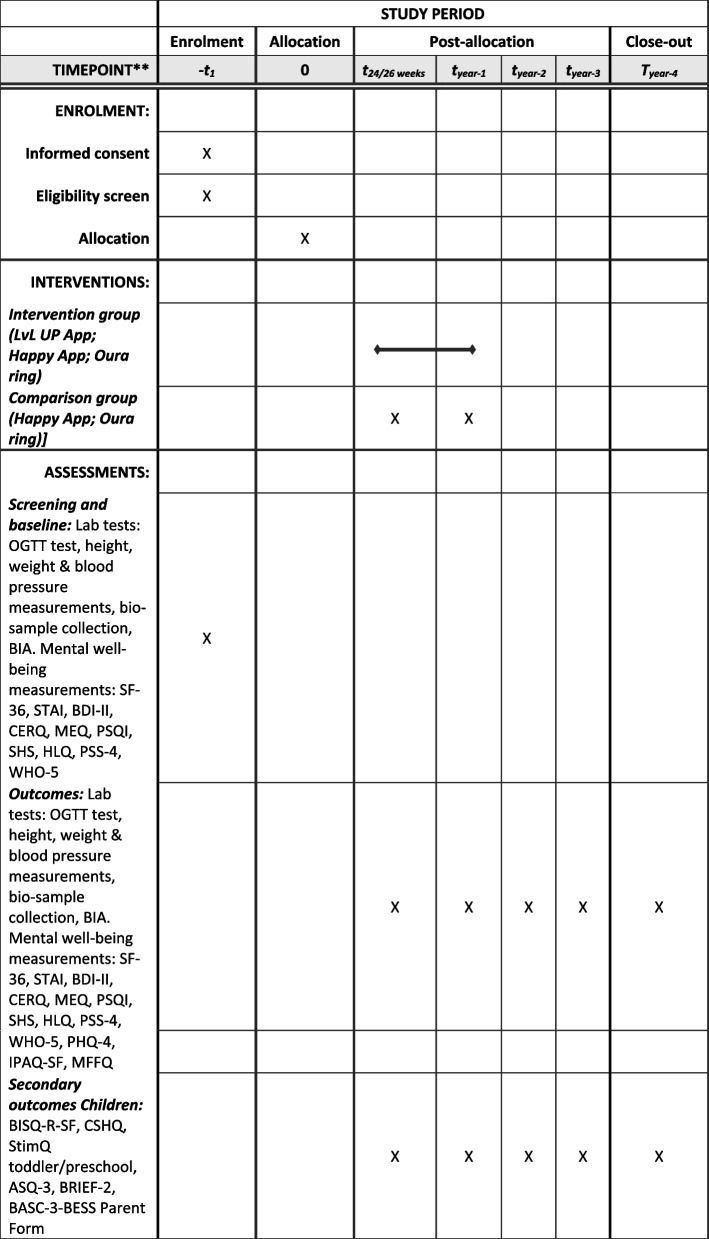
Schedule for enrolment, intervention, and assessments for the trial. OGTT, 2-h 75 g Oral Glucose Tolerance Test; BIA, Bioelectrical impedance analysis, SF-36, short-form health survey; STAI, The Stare-Trait Anxiety Inventory; BDI-II, Beck Depression Inventory; CERQ, Cognitive Emotion Regulation Questionnaire; MEQ, Morningness-Eveningness Questionnaire; PSQI, The Pittsburgh Sleep Quality Index; SHS, Subjective Happiness Scale; HLQ, Health Literacy Questionnaire; PSS-4, Perceived Stress Scale 4; WHO-5, World Health Organization Wellbeing Index; BISQ-R-SF, Brief Infant Sleep Questionnaire-Revised Short Form; CSHQ, Children’s Sleep Habits Questionnaire; StimQ toddler/preschool, Cognitive Home Environment Questionnaire; ASQ-3, Ages & Stages Questionnaires-3; BRIEF-2, Behaviour Rating Inventory of Execute Functions-2; BASC-3-BESS Parent Form, Behavioral and Emotional Screening System; PHQ-4, Patient Health Questionnaire; IPAQ-SF, The International Physical Activity Questionnaire- Short Form; MFFQ, Modified Food Frequency Questionnaire

The participants’ flow for the trial is outlined in Fig. 1. Once participants are identified through one of the recruiting methods, they will be invited to the SICS clinic for screening, in which a consent form will be explained and signed, and OGTT test, height, weight, blood pressure measures, HbA1c (finger proc test) and sociodemographic survey will be completed. Those who are eligible will be randomised to one of the two arms (comparison or intervention group) and will subsequently be asked to take follow-up height, weight and blood pressure measurements, BIA, report the child’s height and weight, fasting blood draw, collection of the buccal swab, saliva, and stool sample, and complete questionnaires for the mother and their child at midpoint (26/27 weeks) visit, and year one visit after randomisation. After the midpoint assessments, additional interventions will be delivered via the HAPPY App to subjects in the intervention group who require further recommendations to improve their BMI or manage their body weight. During the 3 year-followed-up periods, participants will be asked to complete the following: (a) OGTT test; (b) height, weight and blood pressure measurements; (c) BIA; (d) fasting blood draw; (e) collection of the buccal swab, saliva and stool samples; (f) reported child’s height and weight; (g) complete mother and child questionnaires; (h) issue of Oura Ring (8 weeks); and (i) 14-day e-Diary recording in year 2, 3 and 4 visit. The schedule of enrolment, intervention, and assessments for the trial is outlined in Fig. 2.

### Sample size {14}

#### Expected number of participants

The sample size is estimated based on the cumulative diabetes risk identified from the GUSTO study [12]. We assume groups of equal size, with a significance level of 0.05% and a power of 80%. Additionally, we consider a median survival time of 6 years for the non-intervention group (based on GUSTO data), a follow-up period of 3 years, and an expected 50% reduction in the risk of developing T2D for the intervention group (hazard ratio = 0.5). These assumptions yield a cumulative event rate of 0.22 for the comparison group and 0.12 for the intervention group. Considering a 20% censoring rate, we calculate a sample size of 189 for the intervention group and 188 for the comparison group, aiming for a total of 65 events over the study period[63].

### Recruitment {15}

The study protocol was approved by the National Healthcare Group in Singapore, Domain Specific Review Board. There are four avenues of recruitment. The first is contacting participants from a previous pilot study conducted by the same research team in this protocol [64]. The second avenue is recruiting participants using snowball sampling (e.g. friends, family), the third is via electronic flyer invitation through corporate e-mails (National University Healthcare System (NUHS) and Agency for Science, Technology and Research (A*STAR)), and the fourth is advertising on social media (e.g. LinkedIn, Instagram, Facebook advertisements). Interested individuals will contact the study team via phone or e-mail. The study team will explain the study and answer any questions raised*.* Potential participants will be invited to attend a screening visit for informed consent and screening procedures. Those who are screened passed will be scheduled to attend baseline and midpoint visits (26/27 weeks), year 1, and follow-up visit periods in years 2, 3, and 4. All the participants will be able to provide written informed consent.

## Assignment intervention: allocation

### Sequence generation {16a}

A total of 400 women will be stratified and randomised based on prognosis factors to either group 1 (intervention) or group 2 (control) based on 1:1 allocation using R statistical software.

### Concealment mechanism {16b}

The allocation is concealed in that the allocation sequence is automatically generated in R software and simultaneous with the assignment.

### Implementation {16c}

The allocation sequence will automatically be generated in the Block Rand R software at the end of the baseline, and a screen will show participants to which group she has been assigned.

## Assignment of interventions: blinding

### Who will be blinded {17a}

Given the nature of the study design, blinding of participants and research personnel will not be possible. The study team will instruct the participants in the intervention and comparison groups to download the Apps, and each participant will be provided with a unique identification code.

### Procedure for unblinding if needed {17b}

In the case of high depression scores on the BDI, including item 9 (suicidal ideation) and anxiety on the STAI, the participant will be flagged and reported to research coordinators, who will contact the participant to provide additional referral and helpline resources in Singapore. Similarly, scores that reveal a potential developmental delay in the child measured by the ASQ-3 will be notified to the mother, and a referral will be made for further evaluation.

## Data collection and management

### Plans for assessment and collection of outcomes{18a}

The baseline, midpoint visit (26/27 weeks), 1-year visit, and 3-year follow-up assessments will be administered on-site through the Recap platform assisted by research coordinators. The REDCap will be used for all the measurements and will follow complex skip logic developed for this study, based on participants’ responses to reduce respondent burden and increase the completeness and accuracy of responses.

The intervention group randomised to the LvL UP App will collect objective and self-reported scales from the mHealth App throughout the intervention.

### Plans to promote participant retention and complete follow-up {18b}

Participants will be reimbursed for their time, inconvenience and transportation costs as follows: (a) screening visit — S$20; (b) completion of baseline study visit — S$80; (c) completion of midpoint study visit — S$40; (d) completion of year 1 study visit — S$80; (e) completion of level 1 to level 3 digital coaching sessions via LvL UP App (applicable to participants in intervention group) will be a total of S$120 and S$40 per level of completion; (f) completions for all three study visits, participants will be given an additional S$80 as bonus; and (g) completion of year 2, 3 and 4 yearly visit the participants will receive S$80 per visit. The research team will call the participants to remind them to attend the visits.

Additionally, the intervention group will receive reminders from the LvL UP App regarding the day and time of the coaching session and daily random lifehack from any of the pillars.

### Data management {19}

The Singapore Institute for Clinical Sciences (SICS) team will maintain a master dataset for all participants who were referred to the project for recruitment, along with a record of whether they withdrew before or after completing the screening and baseline measurements or after initiating the intervention, were considered ineligible for participation, or were terminated from the study or conducted the study. Protection of participant privacy concerning biological samples and questionnaire data will be achieved by assigning each participant a study identification number and then creating a separate de-identified file. Anonymised data from the participants will be shared between SEC and SICS through a secure AWS server. All data analyses will be carried out with the de-identified data file.

### Confidentiality {27}

Identifiable individual participant information obtained from this study is confidential, and disclosure to non-relevant third parties is prohibited. Participant confidentiality will be further ensured by assigning each participant a unique participant identification code for anonymised data entry and sample identification. All computer entry and networking programs will be done with coded numbers only.

## Statistical methods

### Statistical methods for primary and secondary outcomes {20a}

#### Analysis of aggregate intervention effects

The primary continuous outcome for the onset of diabetes will be analyzed with linear mixed models (LMM) with outcome measurement (at the three follow-up time points) as the dependent variable and based on the intention-to-treatment (ITT) principle.

Multiple linear regressions will determine the relationship between outcome measures and predictors, adjusting for potential confounders. Multiple linear regression analyses will estimate the effects between groups at the end point follow-up. Generalised estimating equations and mixed effect models will be used for longitudinal data analysis. A *p*-value of < 0.05 will be considered statistically significant. Correction for multiple tests will be performed. All statistical analyses will be conducted using the R program or STATA for Windows (Stata Corporation). The treatment effect within and between the two groups will be assessed using the Cohen *d* statistics [65].

### Interim analyses {21b}

Interim analyses will be conducted to examine initial trial efficacy and safety outcomes. Frequencies (percentages), means (standard deviations), or medians (interquartile ranges) will be used to summarise the data collected from questionnaires and surveys. Statistical tests will be performed, e.g. Chi-square or Fisher’s exact test for categorical variables, and two independent sample *t*-tests or ANOVA for continuous variables. Nonparametric tests will be used as deemed fit. For binary outcomes, the strength of associations between independent and dependent variables will be assessed by odds ratio estimated in logistic regression models. Crude associations will be evaluated using univariate models. Subsequently, associations will be evaluated using multivariable models adjusted for potential confounders.

### Methods for additional analyses (e.g. subgroup analyses) {20b}

We will carry out additional analyses that evaluate the effect of the intervention on the children born to the women during the GDM pregnancy with a focus on cognitive and socio-emotional development (ASQ-3, BASC-3-BESS, BRIEF, StimQ) and sleep (BISQ-R-SF, CSHQ) using linear mixed models (LMM) with outcome measurements (baseline and the three follow-up time points) based on intention-to-treat (ITT).

Descriptive statistics will be used to establish the intervention response rate based on the number of coaching sessions completed, logins into the App, and estimated time spent.

### Methods in analysis to handle protocol non-adherence and any statistical methods to handle missing data {20c}

Intention-to-treat analysis will be used to examine trial efficacy. Total scores will be modelled using fixed effects of time, intervention group, interaction between time and intervention group, and a random effect of individual.

### Plans to give access to the full protocol, participant-level data and statistical code {31c}

The anonymized datasets analysed during the current study and statistical code are available from the corresponding author on reasonable request, as is the full protocol.

## Oversight and monitoring

### Composition of the coordinating centre and trial steering committee {5d}

The study team will have overall responsibility for monitoring the integrity of the study and participant safety. The study team will monitor consent procedures, and safety plans prior to study initiation, amendments thereafter, and monitoring study progress, in terms of recruitment and retention of participants, adverse events, and protocol deviations.

### Composition of the data monitoring committee, its role and reporting structure {21a}

The committee comprises research coordinators (RCs), a data manager, co-investigators, and the principal investigator (JGE). RCs are responsible for electronic data capture following the standard operating procedure, and the data manager monitors data collection. The data manager will raise queries with RCs during data monitoring. The data manager prepares data exports for monthly review and reports to the principal investigator and co-investigators.

### Adverse event reporting and harms {22}

The Principal Investigator (PI) (JGE) will manage the data and safety monitoring after participant enrolment. In the event of any adverse events or safety issues, appropriate reporting will be made to DSRB accordingly. Regarding the questionnaires, we have the following monitoring plan: (a)BDI-II: participants who exhibit suicidality ideation (item 9) or have scored 29 or above (severe depression) will be offered a clinical referral. If the participant declines the referral, she will be provided with local helpline contact numbers (Samaritans of Singapore and Institute of Mental Health); (b) STAI: for participants who exhibit high anxiety with a score of 45 or above in the STAI, the study team will provide a local helpline contact number (Singapore Association for Mental Health). Additionally, the PHQ-4 will be completed by the LvL UP App participants (intervention group) with a score of 9 or higher (severe anxiety depression); the LvL UP App will provide information regarding mental health services in Singapore, including local helplines. The study team will follow up with the participant if she wishes to get a clinical referral. Finally, based on the ASQ-3, if a child shows scores suggesting developmental delay, we will discuss with the participant if she has any concerns and would like a referral for clinical follow-up.

Biological samples will be collected through blood tests, which will be analyzed by an accredited lab providing the normal reference range of the lab results. The PI (JGE) will review blood test results. For all abnormal blood test results and incidental findings discovered during the study period, the study PI will determine if the findings are clinically significant and actionable. The study PI will sign a referral letter to seek primary healthcare. Subsequently, a delegated study team member will contact the participant to relay the internal findings on behalf of the study PI. Participants who agree to be re-identified and notified of the internal findings will be contacted at their primary contact number provided in the consent form. The referral letter will be emailed to her. If the participant is uncontactable on her direct contact number, her next of kin listed on the consent form will be contacted. If the participant and next of kin are not contactable after 1 month, the referral letter will be sent directly to the participant’s home address. In the case incidental findings meet the study exclusion criteria, the affected participant will be informed and withdrawn from the study.

For the rest of the study measures/questionnaires, there is no clinical validation available. Hence no applicable management plan will be carried out.

### Frequency and plans for monitoring trial conduct {23}

Study activities, including those related to consent, randomisation, data collection, and intervention, will be monitored on an ongoing basis***.*** The data management team will monitor the data on the Amazon Web Services (AWS) server and REDCap platform. The study team will upload outcome data by the participants at the AWS server monthly.

### Plans for communicating necessary protocol amendments to relevant parties (e.g. trial participants, ethical committees) {25}

Changes to the study protocol will be communicated to the DSRB when required. Deviations from the protocol will be fully documented using a breach report form and it will be updated to the protocol in the clinical trial registry.

### Dissemination plans {31a}

Study results will be disseminated through webinars, written documents, conferences, and research meetings at SICS. We will also prepare scientific reports of the study results for publication in peer-reviewed journals.

## Discussion

This study aims to identify post-GDM women from community settings in Singapore and assess the effectiveness of a holistic lifestyle mHealth intervention for the prevention of T2D and CMDs. It is essential to develop preventative interventions to encourage healthy lifestyle habits, given the high incidence rates of T2D and CMD among women following GDM. This study may provide further support for the effectiveness of preventative interventions that consider the close relationship between physical and psychological health. Holistic lifestyle interventions that promote healthy habits such as maintaining a balanced healthier diet, engaging in regular physical activity, getting adequate sleep, and prioritising mental well-being can work together to produce more effective results [34, 66, 67]. mHealth also offers practical, affordable, and scalable intervention support for individuals adopting and maintaining healthy lifestyles. The evidence has shown that holistic mHealth targeting lifestyle behaviours are available and have shown to be effective [31, 34, 37]. However, few studies have been tested using multicompetent lifestyle using mHealth [34]. This RCT study represents a pioneer attempt to investigate the effectiveness of a holistic lifestyle mHealth intervention focused on the prevention of T2D and CMDs in women with a history of GDM.

mHealth has the potential to transform preventive healthcare, and particularly support post-GDM women who encounter challenges due to fatigue, child-caring responsibilities, and motivation to change their lifestyles. Additionally, we expect that the holistic mHealth proposed will permit to build capacities to disseminate interventions and could have enormous public health implications for the region and serve as a model for implementing preventative interventions in Asia and globally.

## Trial status

DSRB Approval of Protocol Version 1.0; 1/6/2023. Recruitment began 20/10/2023. Recruitment is tentatively scheduled to be completed on 30/10/2024.

### Supplementary Information

Additional file 1: SPIRIT checklist.

## Data Availability

Final de-identified data and trial materials will be publicly available after the trial is concluded.

## Abbreviations

A*STAR: Agency for Science, Technology and Research
AWS: Amazon Web Services
BASC-3-BESS: Behavioral and Emotional Screening System
BCT: Behavioural change techniques
BISQ-R-SF: Brief Infant Sleep Questionnaire-Revised Short Form
BDI-II: Beck Depression Inventory
BRIEF-2: Behaviour Rating Inventory of Execute Functions-2
CBT: Cognitive behavioural therapy
CERQ: Cognitive Emotion Regulation Questionnaire
CMD: Common mental disorders
CSHQ: Children’s Sleep Habits Questionnaire
DSRB: Domain-Specific Review Board
DPP: Diabetes Prevention Program
HLQ: Health Literacy Questionnaire
GDM: Gestational diabetes mellitus
MEQ: Morningness-Eveningness Questionnaire
NUHS: National University Health System
NUS: National University of Singapore
PA: Physical activity
PHQ-4: Patient health questionnaire, 4-item
PI: Principal investigator
PSQI: The Pittsburgh Sleep Quality Index
PSS-4: Perceived Stress Scale 4
PP: Positive psychology
RCT: Randomised control trial
SEC: Singapore ETH-Centre
SHS: Subjective Happiness Scale
SICS: Singapore Institute for Clinical Sciences
SF-36: Short-form health curve
STAI: The State-Trait Anxiety Inventory
StimQ toddler/preschool: Cognitive Home Environment Questionnaire toddler/preschool
T2D: Type 2 diabetes
WHO-5 Wellbeing Index: World Health Organization-5 Wellbeing Index

## References

[1] Federation ID. IDF Diabetes Atlas. 10th ed. Brussels; 2021. https://www.diabetesatlas.org.

[2] Saeedi P, Petersohn I, Salpea P, Malanda B, Karuranga S, Unwin N (2019). Global and regional diabetes prevalence estimates for 2019 and projections for 2030 and 2045: Results from the International Diabetes Federation Diabetes Atlas. Diabetes Res Clin Pract.

[3] Sun J, Hu W, Ye S, Deng D, Chen M (2023). The description and prediction of incidence, prevalence, mortality, disability-adjusted life years cases, and corresponding age-standardized rates for global diabetes. J Epidemiol Glob Health.

[4] Collaborators GOD, Bernabe E, Marcenes W, Hernandez C, Bailey J, Abreu L (2020). Global, regional, and national levels and trends in burden of oral conditions from 1990 to 2017: a systematic analysis for the global burden of disease 2017 study. J Dent Res.

[5] Monod C, Kotzaeridi G, Linder T, Eppel D, Rosicky I, Filippi V (2023). Prevalence of gestational diabetes mellitus in women with a family history of type 2 diabetes in first-and second-degree relatives. Acta Diabetol.

[6] McIntyre HD, Catalano P, Zhang C, Desoye G, Mathiesen ER, Damm P (2019). Gestational diabetes mellitus. Nat Rev Dis Primers.

[7] Li L-J, Huang L, Tobias DK, Zhang C (2022). Gestational Diabetes Mellitus Among Asians–A Systematic Review From a Population Health Perspective. Front Endocrinol.

[8] Kunasegaran T, Balasubramaniam VR, Arasoo VJ, Palanisamy UD, Ramadas A. Gestational diabetes mellitus in Southeast Asia: a scoping review. Int J Environ Res Public Health. 2021;18(3):1272. 10.3390/ijerph18031272. https://www.mdpi.com/1660-4601/18/3/1272. 10.3390/ijerph18031272 PMC7908368 33572656

[9] Hod M, Kapur A, Sacks DA, Hadar E, Agarwal M, Di Renzo GC (2015). The International Federation of Gynecology and Obstetrics (FIGO) Initiative on gestational diabetes mellitus: A pragmatic guide for diagnosis, management, and care. Int J Gynecol Obstet.

[10] Kc K, Shakya S, Zhang H (2015). Gestational diabetes mellitus and macrosomia: a literature review. Ann Nutr Metab.

[11] Vounzoulaki E, Khunti K, Abner SC, Tan BK, Davies MJ, Gillies CL (2020). Progression to type 2 diabetes in women with a known history of gestational diabetes: systematic review and meta-analysis. Bmj..

[12] Chen L-W, Soh SE, Tint M-T, Loy SL, Yap F, Tan KH (2021). Combined analysis of gestational diabetes and maternal weight status from pre-pregnancy through post-delivery in future development of type 2 diabetes. Sci Rep.

[13] Ukke GG, Boyle JA, Reja A, Lee WK, Chen M, Ko MSM (2023). Lifestyle Interventions to Prevent Type 2 Diabetes in Women with a History of Gestational Diabetes: A Systematic Review and Meta-Analysis through the Lens of Health Equity. Nutrients.

[14] Plows JF, Stanley JL, Baker PN, Reynolds CM, Vickers MH (2018). The pathophysiology of gestational diabetes mellitus. Int J Mol Sci.

[15] OuYang H, Chen B, Abdulrahman AM, Li L, Wu N. Associations between gestational diabetes and anxiety or depression: a systematic review. J Diabetes Res. 2021;2021(1):9959779. 10.1155/2021/9959779. https://link.springer.com/article/10.1007/s44197-023-00138-9. 10.1155/2021/9959779 PMC8337159 34368368

[16] Wilson CA, Newham J, Rankin J, Ismail K, Simonoff E, Reynolds R (2020). Is there an increased risk of perinatal mental disorder in women with gestational diabetes? A systematic review and meta-analysis. Diabet Med.

[17] O'Hara MW, Wisner KL (2014). Perinatal mental illness: definition, description and aetiology. Best Pract Res Clin Obstet Gynaecol.

[18] Stein A, Pearson RM, Goodman SH, Rapa E, Rahman A, McCallum M (2014). Effects of perinatal mental disorders on the fetus and child. The Lancet.

[19] Wu Q, Meng Z, Liu Q, Zhang L, Mao B, Wang C (2023). Sleep quality in women with diabetes in pregnancy: a single-center retrospective study. BMC Pregnancy Childbirth.

[20] Itani O, Jike M, Watanabe N, Kaneita Y (2017). Short sleep duration and health outcomes: a systematic review, meta-analysis, and meta-regression. Sleep Med.

[21] Saadati F, Sehhatiei Shafaei F, Mirghafourvand M (2018). Sleep quality and its relationship with quality of life among high-risk pregnant women (gestational diabetes and hypertension). J Matern Fetal Neonatal Med.

[22] Goveia P, Cañon-Montañez W, Santos DdP, Lopes GW, Ma RC, Duncan BB (2018). Lifestyle intervention for the prevention of diabetes in women with previous gestational diabetes mellitus: a systematic review and meta-analysis. Front Endocrinol.

[23] Zhang C, Tobias DK, Chavarro JE, Bao W, Wang D, Ley SH (2014). Adherence to healthy lifestyle and risk of gestational diabetes mellitus: prospective cohort study. Bmj..

[24] Tuomilehto J, Lindström J, Eriksson JG, Valle TT, Hämäläinen H, Ilanne-Parikka P (2001). Prevention of type 2 diabetes mellitus by changes in lifestyle among subjects with impaired glucose tolerance. N Engl J Med.

[25] Zakaria H, Abusanana S, Mussa BM, Al Dhaheri AS, Stojanovska L, Mohamad MN (2023). The Role of Lifestyle Interventions in the Prevention and Treatment of Gestational Diabetes Mellitus. Medicina.

[26] Huang S, Magny-Normilus C, McMahon E, Whittemore R (2022). Systematic review of lifestyle interventions for gestational diabetes mellitus in pregnancy and the postpartum period. J Obstet Gynecol Neonatal Nurs.

[27] Lie M, Hayes L, Lewis-Barned N, May C, White M, Bell R (2013). Preventing type 2 diabetes after gestational diabetes: women's experiences and implications for diabetes prevention interventions. Diabet Med.

[28] Pagoto S. The current state of lifestyle intervention implementation research: where do we go next? Transl Behav Med. 2011;1(3):401–5. 10.1007/s13142-011-0071-x. https://academic.oup.com/tbm/article-abstract/1/3/401/4562980?redirectedFrom=fulltext. 10.1007/s13142-011-0071-x PMC3717623 24073065

[29] Lim S, Tan A, Madden S, Hill B (2019). Health professionals' and postpartum women's perspectives on digital health interventions for lifestyle management in the postpartum period: a systematic review of qualitative studies. Front Endocrinol.

[30] Moschonis G, Siopis G, Jung J, Eweka E, Willems R, Kwasnicka D (2023). Effectiveness, reach, uptake, and feasibility of digital health interventions for adults with type 2 diabetes: a systematic review and meta-analysis of randomised controlled trials. The Lancet Digital Health.

[31] Mair JL, Salamanca-Sanabria A, Augsburger M, Frese BF, Abend S, Jakob R (2023). Effective behavior change techniques in Digital health interventions for the prevention or management of noncommunicable diseases: an umbrella review. Ann Behav Med.

[32] Keller R, Hartmann S, Teepe GW, Lohse K-M, Alattas A, Tudor Car L (2022). Digital behavior change interventions for the prevention and management of type 2 diabetes: systematic market analysis. J Med Internet Res.

[33] Andersson G, Carlbring P, Titov N, Lindefors N (2019). Internet interventions for adults with anxiety and mood disorders: a narrative umbrella review of recent meta-analyses. Can J Psychiatr.

[34] Zheng S, Edney SM, Goh CH, Tai BC, Mair JL, Castro O, et al. Effectiveness of holistic mobile health interventions on diet, and physical, and mental health outcomes: a systematic review and meta-analysis. EClinicalMedicine. 2023;66. 10.1016/j.eclinm.2023.102309. https://www.thelancet.com/journals/eclinm/article/PIIS2589-5370(23)00486-8/fulltext. 10.1016/j.eclinm.2023.102309 PMC10694579 38053536

[35] Castro O, Mair JL, Salamanca-Sanabria A, Alattas A, Keller R, Zheng S (2023). Development of “LvL UP 1.0”: a smartphone-based, conversational agent-delivered holistic lifestyle intervention for the prevention of non-communicable diseases and common mental disorders. Front Digit Health.

[36] Hofmann SG, Asnaani A, Vonk IJ, Sawyer AT, Fang A (2012). The efficacy of cognitive behavioral therapy: A review of meta-analyses. Cogn Ther Res.

[37] Zheng S, Edney SM, Mair JL, Kowatsch T, Castro O, Salamanca-Sanabria A, et al. Holistic mHealth interventions for the promotion of healthy ageing: protocol for a systematic review. BMJ Open. 2023;13(5):e066662. 10.1136/bmjopen-2022-066662. 10.1136/bmjopen-2022-066662 PMC10163532 37130675

[38] Löwe B, Wahl I, Rose M, Spitzer C, Glaesmer H, Wingenfeld K (2010). A 4-item measure of depression and anxiety: validation and standardization of the Patient Health Questionnaire-4 (PHQ-4) in the general population. J Affect Disord.

[39] Craig C, Marshall A, Sjostrom M, Bauman A, Lee P, Macfarlane D (2017). International physical activity questionnaire-short form. J Am Coll Health.

[40] Lukic YX, Teepe GW, Fleisch E, Kowatsch T (2022). Breathing as an input modality in a gameful breathing training app (breeze 2): development and evaluation study. JMIR Serious Games.

[41] Borghouts J, Eikey E, Mark G, De Leon C, Schueller SM, Schneider M (2021). Barriers to and facilitators of user engagement with digital mental health interventions: systematic review. J Med Internet Res.

[42] Jakob R, Harperink S, Rudolf AM, Fleisch E, Haug S, Mair JL (2022). Factors Influencing Adherence to mHealth Apps for Prevention or Management of Noncommunicable Diseases: Systematic Review. J Med Internet Res.

[43] Wang Y-P, Gorenstein C (2013). Psychometric properties of the Beck Depression Inventory-II: a comprehensive review. Braz J Psychiatr.

[44] Spielberger CD. State‐trait anxiety inventory. The Corsini Encyclopedia of Psychology. 2010:1. 10.1002/9780470479216.corpsy0943.

[45] Jermann F, Van der Linden M, d'Acremont M, Zermatten A (2006). Cognitive emotion regulation questionnaire (CERQ). Eur J Psychol Assess.

[46] Lyubomirsky S, Lepper HS (1999). A measure of subjective happiness: Preliminary reliability and construct validation. Soc Indic Res.

[47] Sischka PE, Costa AP, Steffgen G, Schmidt AF (2020). The WHO-5 well-being index–validation based on item response theory and the analysis of measurement invariance across 35 countries. Journal of Affective Disorders Reports.

[48] Chan SF, La Greca AM (2020). Perceived stress scale (PSS).

[49] Ware JE (2000). SF-36 health survey update. Spine.

[50] Osborne RH, Batterham RW, Elsworth GR, Hawkins M, Buchbinder R (2013). The grounded psychometric development and initial validation of the Health Literacy Questionnaire (HLQ). BMC Public Health.

[51] Adan A, Almirall H (1991). Horne & Östberg morningness-eveningness questionnaire: A reduced scale. Personality Individ Differ.

[52] Smyth C (1999). The Pittsburgh sleep quality index (PSQI).

[53] Mendelsohn A, Dreyer B, Tamis-LeMonda C, Ahuja P (1999). Validity of StimQ, a scale for assessing the cognitive home environment. J Dev Behav Pediatr.

[54] Cates CB, Roby E, Canfield CF, Johnson M, Raak C, Weisleder A (2023). Validation of the StimQ2: A parent-report measure of cognitive stimulation in the home. PLoS ONE.

[55] Squires J, Bricker DD, Twombly E. Ages & stages questionnaires. Baltimore: Paul H. Brookes; 2009. https://aaimsschool.com/uploads/3/5/3/0/35304913/printable_asq_developmental_guide_1month_-5.5years_old.pdf.

[56] LeJeune B, Beebe D, Noll J, Kenealy L, Isquith P, Gioia G (2010). Psychometric support for an abbreviated version of the Behavior Rating Inventory of Executive Function (BRIEF) Parent Form. Child Neuropsychol.

[57] Reynolds CR, Kamphaus RW, Vannest KJ. BASC3: Behavior assessment system for children. San Antonio: PscyhCorp; 2015. https://link.springer.com/referenceworkentry/10.1007/978-3-319-56782-2_1524-2.

[58] Mindell JA, Gould RA, Tikotzy L, Leichman ES, Walters RM (2019). Norm-referenced scoring system for the brief infant sleep questionnaire–revised (BISQ-R). Sleep Med.

[59] Bonuck KA, Goodlin-Jones BL, Schechter C, Owens J (2017). Modified Children's sleep habits questionnaire for behavioral sleep problems: A validation study. Sleep Health.

[60] (HPB) HPB. My healthy plate [Available from: https://www.healthhub.sg/programmes/nutrition-hub/eat-more?utm_source=google&utm_medium=paid-search&utm_campaign=fy23-nl-edsh-ao&utm_content=fy23-nl-edsh-ao_google_paid-search_my-healthy-plate&gclid=Cj0KCQiAm4WsBhCiARIsAEJIEzW-T5XOZPkMBnn5v33wOHwatbKpGVTTx6s-l4_oIW5wrdabjmf4RhoaAjoCEALw_wcB#my-healthy-plate.

[61] Craig CL, Marshall AL, Sjöström M, Bauman AE, Booth ML, Ainsworth BE (2003). International physical activity questionnaire: 12-country reliability and validity. Med Sci Sports Exerc.

[62] Lee PH, Macfarlane DJ, Lam TH, Stewart SM (2011). Validity of the international physical activity questionnaire short form (IPAQ-SF): A systematic review. Int J Behav Nutr Phys Act.

[63] Schoenfeld DA (1983). Sample-size formula for the proportional-hazards regression model. Biometrics..

[64] Liew SJ, Soon CS, Chooi YC, Tint MT, Eriksson JG. A holistic approach to preventing type 2 diabetes in Asian women with a history of gestational diabetes mellitus: a feasibility study and pilot randomized controlled trial. Front Clin Diabetes Healthc. 2023;4:1251411. 10.3389/fcdhc.2023.1251411. 10.3389/fcdhc.2023.1251411 PMC10569025 37841647

[65] Cohen, J. Statistical Power Analysis for the Behavioral Sciences. 2nd ed. Routledge; 1988. 10.4324/9780203771587. eBook ISBN 9780203771587.

[66] Wong VW-H, Ho FY-Y, Shi NK, Sarris J, Ng CH, Tam OKY (2022). Lifestyle medicine for anxiety symptoms: A meta-analysis of randomized controlled trials. J Affect Disord.

[67] Wong VW-H, Ho FY-Y, Shi N-K, Sarris J, Chung K-F, Yeung W-F (2021). Lifestyle medicine for depression: a meta-analysis of randomized controlled trials. J Affect Disord.

